# The single-cell chromatin landscape in gonadal cell lineage specification

**DOI:** 10.1186/s12864-024-10376-1

**Published:** 2024-05-13

**Authors:** Hoi Ching Suen, Fanghong Ou, Kai-kei Miu, Zhangting Wang, Wai-yee Chan, Jinyue Liao

**Affiliations:** 1 Developmental and Regenerative Biology Program, School of Biomedical Sciences, Faculty of Medicine, The Chinese University of Hong Kong, Shatin, Hong Kong SAR China; 2 Department of Chemical Pathology, Faculty of Medicine, The Chinese University of Hong Kong, Shatin, Hong Kong SAR China

**Keywords:** Gonad development, Sex determination, scATAC-seq, Chromatin accessibility, Regulatory genomics, Single-cell biology, Single-cell sequencing

## Abstract

Gonad development includes sex determination and divergent maturation of the testes and ovaries. Recent advances in measuring gene expression in single cells are providing new insights into this complex process. However, the underlying epigenetic regulatory mechanisms remain unclear. Here, we profiled chromatin accessibility in mouse gonadal cells of both sexes from embryonic day 11.5 to 14.5 using single-cell assay for transposase accessible chromatin by sequencing (scATAC-seq). Our results showed that individual cell types can be inferred by the chromatin landscape, and that cells can be temporally ordered along developmental trajectories. Integrative analysis of transcriptomic and chromatin-accessibility maps identified multiple putative regulatory elements proximal to key gonadal genes *Nr5a1*, *Sox9* and *Wt1*. We also uncover cell type-specific regulatory factors underlying cell type specification. Overall, our results provide a better understanding of the epigenetic landscape associated with the progressive restriction of cell fates in the gonad.

**Supplementary Information:**

The online version contains supplementary material available at 10.1186/s12864-024-10376-1.

## Introduction

Gonadal differentiation has a decisive influence on sexual development. Defects in gonadal development cause Disorders of sex development (DSD), conditions with ambiguous genitalia, impaired steroid hormone production, reduced or null fertility, partial to complete sex reversal, and severe psychological pressure. The dysfunction of the gonadal system is also implicated in fertility problems, cancer, and polycystic ovary syndrome [8, 27].

Bipotential gonads, also known as genital ridges, are paired structures raised from proliferating coelomic epithelium (CE) on the ventromedial surface of the mesonephros at about four weeks post-coitum in human or embryonic day (E) 9.5 in mice [40, 52, 62]. Lineage progression in the gonads of both XX and XY embryos contains two major somatic cell populations—the supporting cells and the steroidogenic cells—both of which are raised from WT1- and NR5A1-positive progenitors [28, 33]. Supporting-like cells (SLCs), a newly discovered cell type, are the first somatic cell lineage to be specified in the bipotential gonad at E10.5, which have been shown to contribute to the formation of the rete testis and rete ovarii, as well as the pool of Sertoli and pre-granulosa cells [42]. In XY mouse gonads, Sertoli cell lineage is specified by the expression of *Sry* at around E10 to E10.5, which in turn upregulates *Sox9* and leads to the formation of Sertoli cell precursors at E11.5 [3, 31]. Some somatic progenitors maintain their cellular states at this stage and differentiate into interstitial precursor cells later, which keep differentiating from E12.5 to E15.5 to produce sufficient steroidogenic fetal Leydig cells for male reproductive tract development [1, 2, 5, 9, 38, 64]. In XX mouse gonads, the -KTS alternatively spliced isoform of Wt1 has been identified as a key determinant of female sex determination [26]. The somatic progenitor cells commit to supporting pre-granulosa cells or stromal progenitor cells. Embryonic granulosa cell recruitment happens between E11.5 and E14.5, contributing to the medullary follicles that are activated at birth [45]. The stromal progenitor cells remain undifferentiated in the fetal ovary and most of them transform into the steroidogenic theca cells when receiving Hedgehog signaling from granulosa cells near the time of birth [52]. Sex determination of the somatic cells further instruct germ cell differentiation as gonocytes in XY or oocytes in XX gonads [53].

With the advance of single cell sequencing technology, the gene expression profiles of sex-specific gonadal cell types in human, mouse, and chicken embryos have been well characterized at the single-cell level [18, 60, 61, 63]. Despite the increasing insights into gene functions in gonadal development, the underlying mechanism of most gonadal disorders still cannot be explained clearly. Understanding of gonadal development from an epigenetic perspective is important. One well-documented example is distal enhancers of haploinsufficient gene *SOX9* related to sex reversal [12]. Different from scRNA-seq, single-cell transposase accessible chromatin sequencing (scATAC-seq) uses Tn5 transposase to cleave and tag open DNA regions with sequencing adaptors in a single-cell droplet, thus providing the chromatin accessibility landscape of the whole genome. scATAC-seq is an effective tool for uncovering cell type-specific active transcription factors and associating genetic variants with disease causal cell types [11, 14].

Here, we characterize the cell type-specific epigenetic regulatory network and cellular differentiation trajectory of both sexes during mouse gonadal development using scATAC-seq. Using these knowledge and resources, we are able to gain a deeper understanding of epigenetic regulation underlying gonad development and lay the foundation for diagnosing unexplained gonadal disorders and designing effective therapies and in vitro gametogenesis in the future.

## Results

### Single-cell ATAC-seq revealed cellular heterogeneity in the XX and XY gonads

**Fig. 1:**
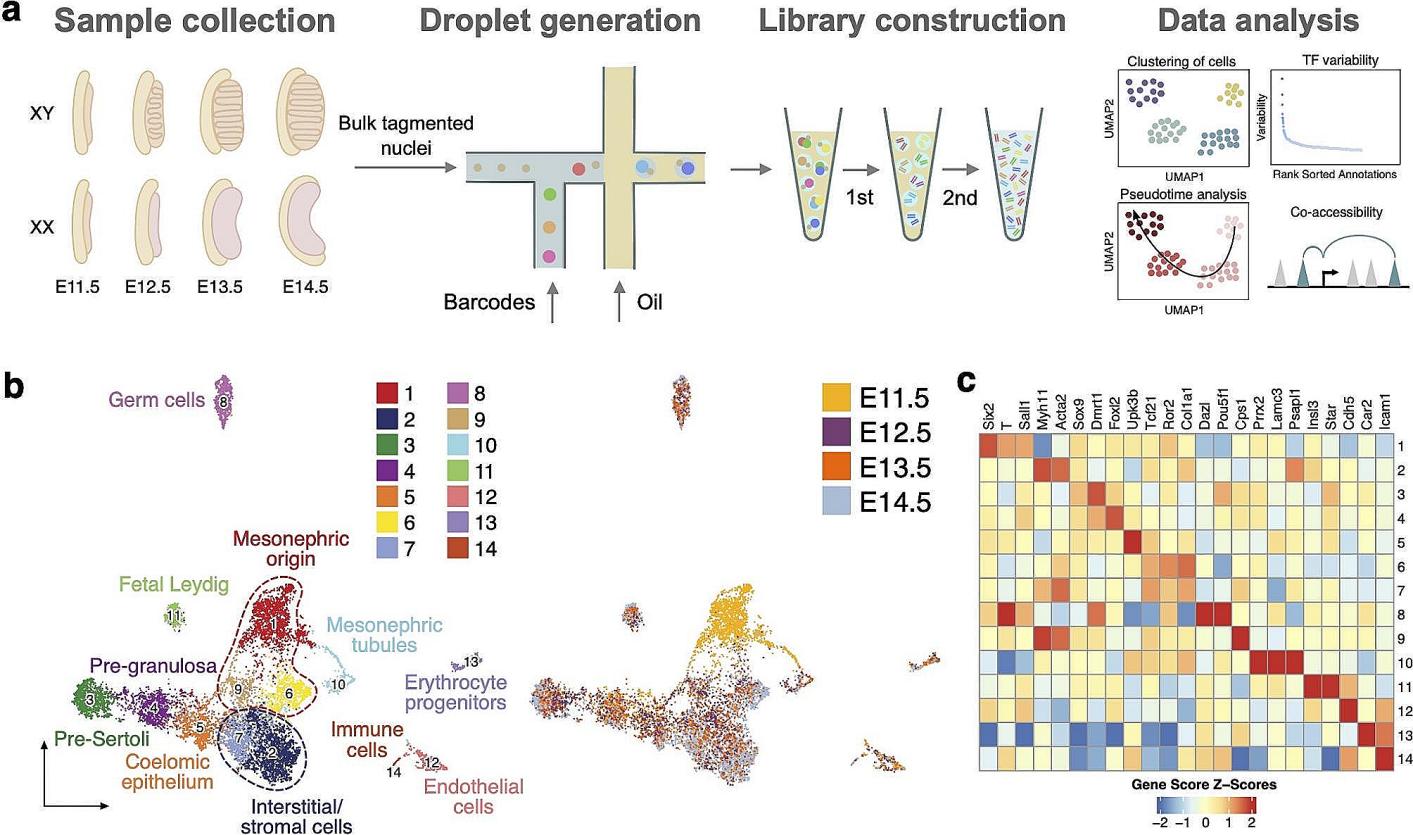
Single-cell ATAC-seq revealed cellular heterogeneity in the XX and XY gonads. (**a**) The workflow of gonad sample collection and scATAC-seq to measure single nuclei accessibility on BioRad SureCell ATAC-seq platform. (**b**) UMAP representation of all cells captured from all four time points. Cells are colored by annotated cell types (left) and time points (right). (**c**) Heatmap showing the gene score of selected cell type marker genes

To resolve heterogeneous cell populations and delineate the chromatin dynamics in gonads during gonad development, we profiled the chromatin accessibility landscapes of female and male mouse gonads at four time points (E11.5, E12.5, E13.5, and E14.5). We dissected the gonad/mesonephros complex and gently removed the mesonephros (except for E11.5). The morphology of the gonad/mesonephros complex was cross-referenced with previous morphological studies of gonad development to ensure the collection of gonads at the appropriate timing [37]. We then applied scATAC-seq using the BioRad SureCell platform (Fig. 1a). Altogether, we profiled the chromatin accessibility in 9494 individual cells after stringent quality control and heterotypic doublet removal (Supplementary Figure S1, Supplementary Table S1). Initial clustering by SnapATAC revealed 14 clusters, with various proportions from E11.5 to E14.5 (Fig. 1b and Supplementary Figure S2a). The sex of the cells was determined in silico by quantifying the reads mapped to the Y chromosome and normalizing to the sequencing depth to give the chrY score (Supplementary Figure S2b-c). Notably, Cluster 3 had a high chrY score, whereas Clusters 4 and 6 had a low chrY score. The remaining clusters exhibited a modest chrY score due to a mix of male and female cells. The clusters will be referred to by cluster number (0 to 14) and, where possible, are also named by their deduced cell types as described below.

We inferred the gene activity scores (which predict gene expression) compiled from chromatin accessibility signals within the gene body and promoter [25]. We then conducted label transfer from a recently published scRNA-seq reference dataset on mouse gonadal cells based on the coordination of gene score from scATAC-seq and gene expression levels from scRNA-seq [42]. Cellular identities were further confirmed by leveraging previous knowledge of the expression status of genes relevant to gonadal development, which were determined by transcriptomic and lineage tracing studies [18, 30, 43, 56, 61]. These include *Six2* in mesonephric cells primarily from E11.5 (Cluster 1), *Myh11* and *Acta2* in interstitial and stromal cells (Clusters 2 and 7), *Sox9* and *Dmrt1* in pre-Sertoli cells (Cluster 3), *Foxl2* in pre-granulosa cells (Cluster 4), *Upk3b* in coelomic epithelium (Cluster 5), *Dazl* and *Oct4* (*Pou5f1*) in germ cells (Cluster 8), *Insl3* and *Star* in fetal Leydig cells (Cluster 11), *Cdh5* in endothelial cells (Cluster 12), *Car2* in erythrocyte progenitors (Cluster 13), as well as *Icam1* in immune cells (Cluster 14) (Fig. 1c).

Taken together, these results show that scATAC-seq is capable of distinguishing all the major cell types in gonads.

### A high-resolution cis-regulatory atlas of the developing mouse gonad

**Fig. 2:**
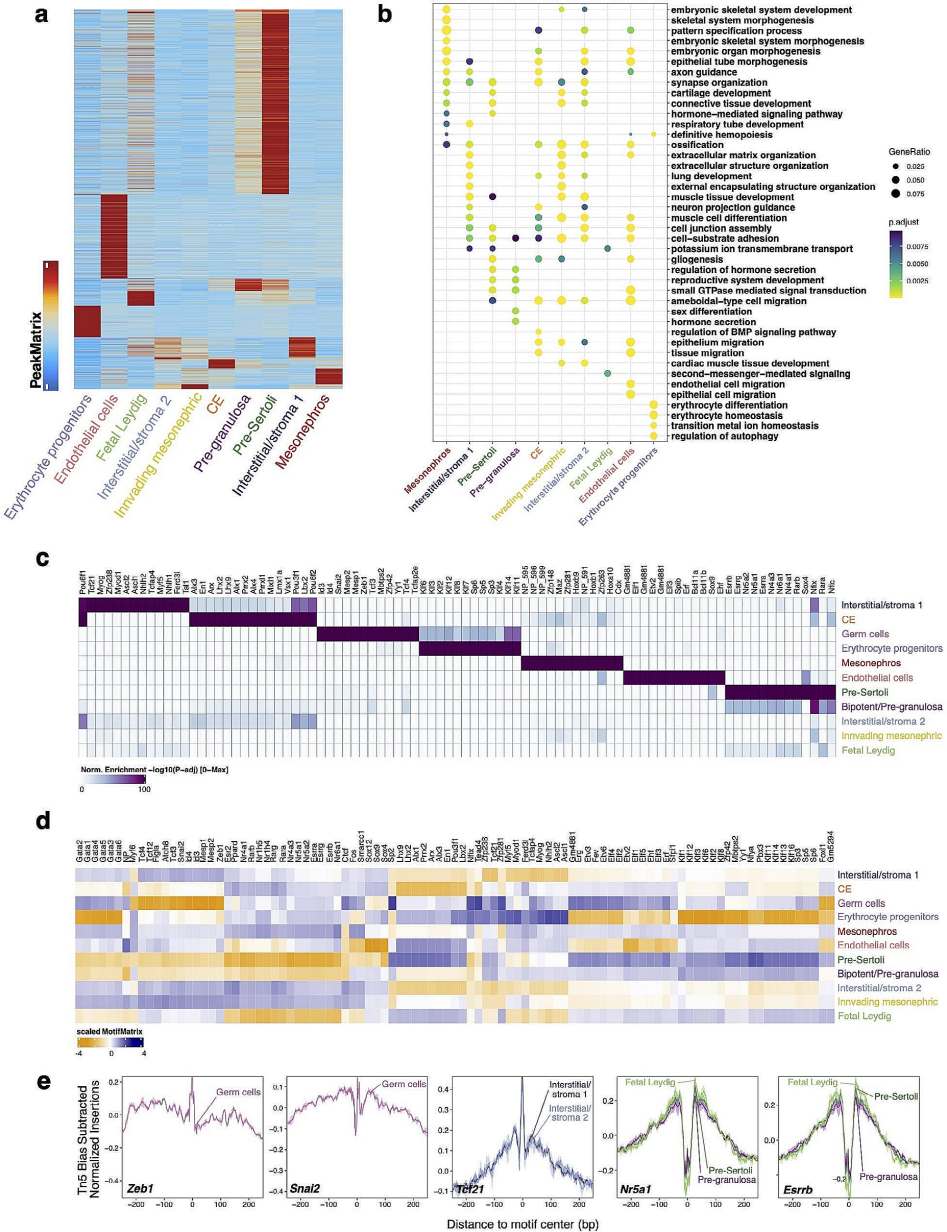
A high-resolution cis-regulatory atlas of the developing mouse gonad. (**a**) Heatmap showing 101,160 marker peaks across cell types (FDR < = 0.1 & Log2FC > = 0.5). (**b**) Top results from the Gene Ontology (GO) enrichment test showing the terms associated with cell type-specific differential accessibility regions (DARs) by associating them to the neighboring gene. (**c**) Heatmap showing the transcription factor (TF) motifs enriched in cell type-specific DARs as shown in Fig. 2a. (**d**) Heatmap showing the chromVAR deviations of top 100 variable TF motifs in each cell type. (**e**) TF footprints (average ATAC-seq signal around predicted binding sites) for selected TFs

To further explore our data, we examined the genomic regions with cell-type differential chromatin accessibilities. We first identified confident ATAC-seq peaks in each cluster using MACS2, and then we combined these peaks to generate a peak set that depicted the entire epigenetic diversity (*n* = 186,471 peaks). We discovered 101,160 differential accessibility regions (DARs) using Wilcoxon testing, taking into account the bias of transcription start site (TSS) enrichment and the number of unique fragments per cell (FDR ≤ 0.1 and log2FC ≥ 0.5) (Fig. 2a, Supplementary Table S2). Next, we associated these DARs with their neighboring genes and performed GO analysis (Fig. 2b). Notably, the Cluster 5 CE progenitor cells showed specific accessible loci related to genes of WNT and BMP signaling pathway. The Cluster 12 endothelial cells showed enrichment of GO terms related to small GTPase mediated signal transduction. The Cluster 4 pre-granulosa cells showed increased accessibility in regions associated with hormone secretion and transport.

Our scATAC-seq data allowed us to probe the specific transcriptional regulatory mechanisms of each cell type. We employed two complementary methods to assess TF motif activity: TF motifs enriched in the cell type-specific DARs (Fig. 2c) and chromVAR deviations (Fig. 2d). chromVAR predicts enrichment of TF activity on a per-cell basis by calculating accessibility deviation across multiple conditions for each TF motif. Using hierarchical clustering of calculated deviations of the top 100 most variable TFs, we identified TFs specific to particular cell subpopulations (Fig. 2d). Overlapping TF bindings identified by both methods signify confident TFs, including known TFs in gonad development such as TCF21 in interstitial progenitors and NR5A1 in fetal Leydig cells (Fig. 2c and d). Additionally, CE cells exhibited enrichment for binding motifs associated with ARX and LHX9, known regulators of Wnt signaling and gonad development. Top enriched TF motifs in pre-Sertoli cells included members of the ESRR, RAR, and SOX families. Notably, SOX4 has a well-described role in male development [65]. We also observed the enrichment of ZEB1 and SNAI2 in germ cells, as well as ETV2 and ELF1 in endothelial cells (Fig. 2c and d). The DNA regions occupied by TF binding are protected from transposition by Tn5, which can be visualized by plotting the ‘footprint’ pattern of each TF as the local chromatin accessibility surrounding the motif midpoint. Footprint occupancy of selected cell type-dependent TFs validated the robustness of our approach (Fig. 2e).

### Chromatin accessibility landscape during somatic cell lineage specification

**Fig. 3:**
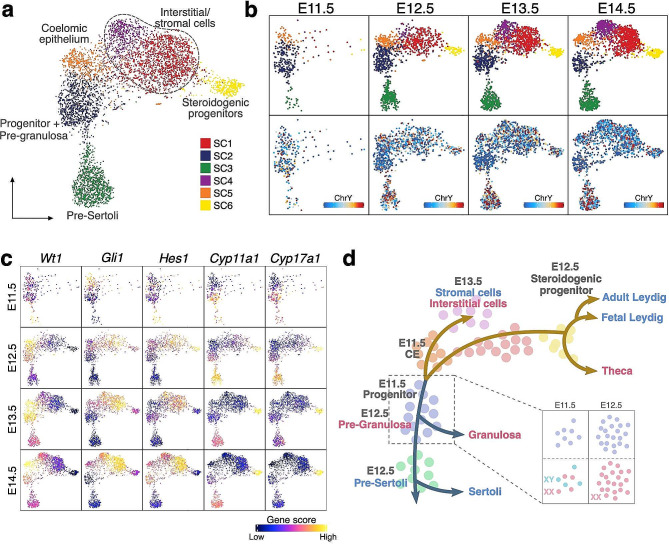
Chromatin accessibility landscape during somatic cell lineage specification. (**a**) UMAP representation of gonadal somatic cells captured from all four time points. Cells are colored by cell clusters. (**b**) UMAP representation of gonadal somatic cells of the four time points. Cells are colored by cell clusters (top panel) and ChrY score (bottom panel). (**c**) UMAP representations with somatic cells colored by the gene score of specific genes at four time points. (**d**) Model of somatic cell lineage specification during sex determination. All somatic gonadal cells are derived from progenitors at coelomic epithelium (CE) at E11.5. For the supporting cell lineage, pre-Sertoli and pre-granulosa cells, deriving from a group of progenitors, start to emerge at E12.5. For the steroidogenic lineage, steroidogenic progenitors emerge at E12.5 and later differentiate to Leydig or theca cell lineages. Interstitial and stromal cells start to be abundant at around E13.5

Although somatic cell commitment has been characterized in depth based on scRNA-seq profiling [18, 61, 63], the underlying chromatin accessibility dynamics remain unclear. Therefore, we tracked the temporal events associated with cell fate progression using our scATAC-seq data. We excluded mesonephric cells, germ cells, endothelial cells and cells from the immune system. We also excluded Clusters 6 and 9 due to their possible mesonephric origin. Re-clustering of these cells generated six clusters, namely somatic clusters (SC) 1 to 6 (Fig. 3a).

At E11.5, somatic cells were already heterogeneous at the chromatin level as three distinct developmental populations emerged (SC2, SC3 and SC5) (Fig. 3b). SC5 cells (shown as Cluster 5 in Fig. 1b) were CE-derived progenitor cells (including gonadal surface epithelium) that showed a high gene score of *Wt1* (Figs. 1c and 3c). They were predisposed to develop into supportive cell lineages in SC2 and SC3, and the XY interstitial or XX stromal cells in SC1 and SC4 at later time points (Fig. 3d). We regarded SC2 cells at E11.5 as progenitors of the “supporting cell lineage”, which was established prior to sex determination (Fig. 3b and d). The absence of any discernible pattern in the scattering of XX and XY cells in UMAP indicated that the chromatin accessibility landscapes in male and female supporting cell progenitors were highly comparable. This similarity may facilitate equitable access to promoters and regulatory elements of sex-determining genes in both sexes, aligning with findings from previous ATAC-seq studies (Garcia-Moreno et al., 2019). SC3 (pre-Sertoli) cells were already apparent by E11.5, indicating that the XY gonad was not fully undifferentiated at this time point, at least at the chromatin level. This also reflected the rapid chromatin accessibility remodeling upon activation of the Sertoli cell fate at this developmental stage. On the contrary, pre-granulosa cells were initially located near the supporting cell progenitor cluster until a slight separation occurred after E13.5, as depicted in the UMAP representation (Fig. 3b). This observation suggests that pre-granulosa cells maintain the progenitor chromatin accessibility landscape for a longer time than pre-Sertoli cells (Fig. 3d). During E11.5 to E12.5, these cell populations expanded, concurrent with the emergence of SC1 and SC6, which were both a mixture of male and female cells based on the ChrY score (Fig. 3b). SC1 represented the interstitial (XY) or stromal (XX) progenitors as they showed increased accessibility at *Gli1* and *Hes1* and loss of accessibility at *Wt1* (Fig. 3c). Some of the cells began to specify their identity as potential steroidogenic cell precursors (SC6), which showed higher gene scores for *Cyp17a1* and *Cyp11a1* (Fig. 3c). Between E12.5 and E13.5, another cell population developed, corresponding to XY interstitial and XX stromal cells (SC4). After E13.5, all cells continued to mature with progressive chromatin accessibility changes.

### Integration of scRNA-seq identified cis-regulatory elements of cell type-specific genes and positive TF regulators

To identify cis-regulatory elements (CREs) of transcription that are linked to somatic lineage-specific gene expression, we reanalyzed, annotated and integrated published scRNA-seq data of mouse gonads from E11.5 to E13.5 [58]. We performed label transfer from this reference scRNA-seq dataset to our scATAC-seq data (Supplementary Figure S4a-c). We then linked the distal peaks to genes in cis, based on the coordination of chromatin accessibility information from scATAC-seq and gene expression levels from scRNA-seq (peak-to-gene links). To demonstrate the utility of peak-to-gene linkage analysis in discovering potential enhancers, we examined the regions upstream of the *Sox9* gene. We found that the Enh13 enhancer region, previously identified in mouse and overlapping within the human XYSR enhancer, showed stronger accessibility in pre-Sertoli cells when compared with other clusters [12, 23]. More importantly, Enh13 was also identified as a significant region linked to the *Sox9* gene with a significant correlation (correlation = 0.62, FDR = 2.09727e-52) (Supplementary Figure S4d). This result highlights the potential of our resource to identify candidate enhancers for genes of interest in the field of reproductive biology.

**Fig. 4:**
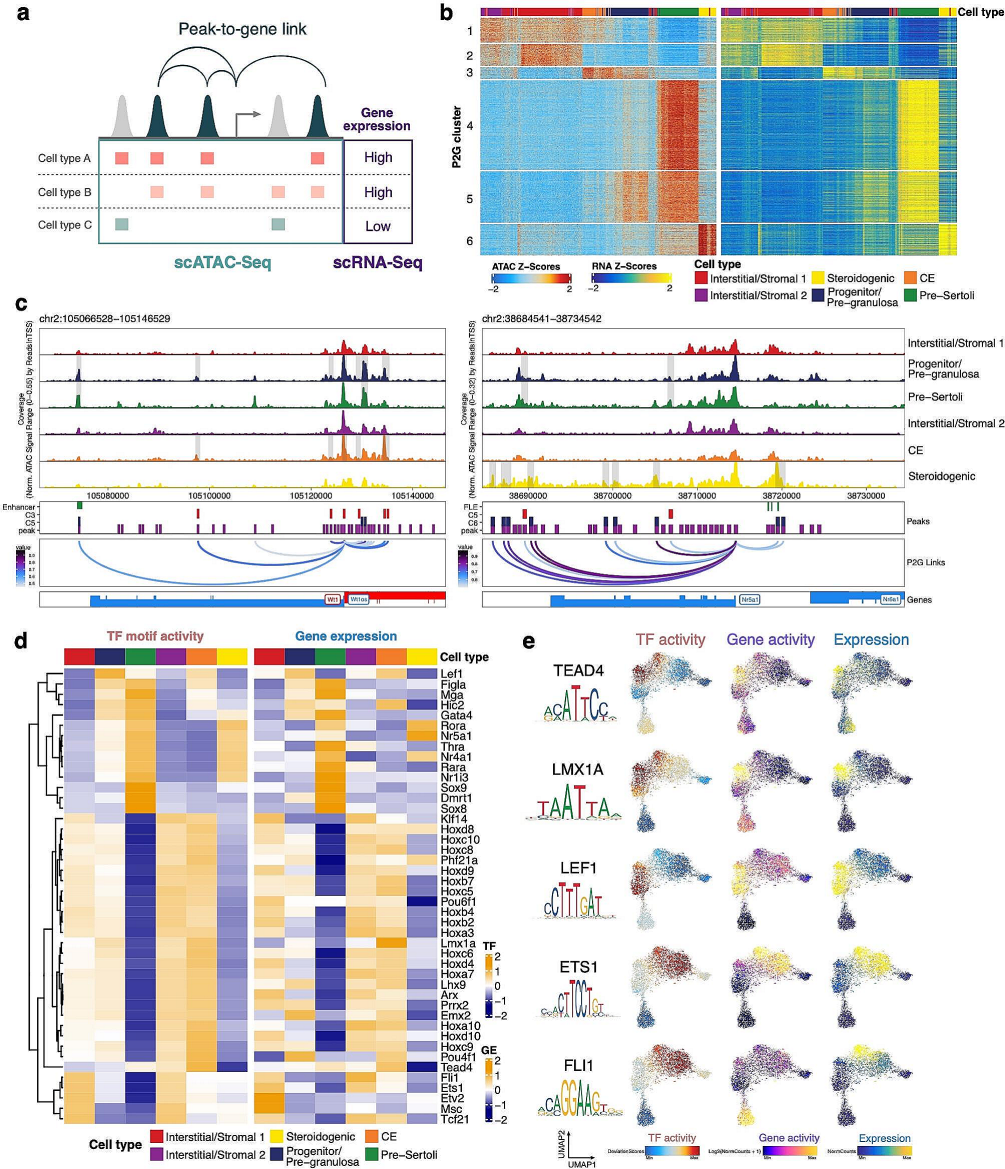
Integration of scRNA-seq identified cis-regulatory elements of somatic cell type-specific genes and positive transcription factor regulators. (**a**) Schematic for identifying significant peak-to-gene linkages by correlating accessible peaks (scATAC-seq data) to gene expression (integrating scATAC seq data and scRNA-seq data). (**b**) Heatmaps showing transcription factor (TF) motif activity (left) and gene expression (right) of 30,561 peak-to-gene linkages across somatic cell types. (**c**) Aggregated scATAC-seq profiles showing peak-to-gene links to the *Wt1* locus overlapped with known enhancer regions (left) and peak-to-gene links to the *Nr5a1* locus overlapped with known fetal Leydig enhancer (FLE) regions (right). (**d**) Heatmaps showing differential TF motif activity (left) and gene activity (right) of positive TF regulators across somatic cell clusters (correlation > 0.3, adjusted p-value < 0.05). (**e**) UMAP embedding of TF chromVAR deviations (left), gene activity scores (middle) and gene expression (right) for selected TFs

We then systematically identified 30,561 peak-to-gene (P2G) links (correlation > 0.5, FDR < 0.01) by correlating ATAC peak accessibility within 250 kb of the gene promoter with the mRNA expression of the gene (Fig. 4a). We performed clustering analysis to identify the cell type-specific P2G links: Clusters (C) 1 and 2, interstitial/stromal progenitors; C3, CE; C4, pre-Sertoli cells; C5, pre-Sertoli/pre-granulosa cells; and C6, steroidogenic progenitors (Fig. 4b). We validated that the enrichments of gene ontologies (GO) for each P2G cluster matched the functions of the respective cell types (Supplementary Figure S5). It is reassuring that this approach discovered previously reported functionally relevant regulatory regions, including enhancers for *Nr5a1* and *Wt1* (discussed in more detail below) [39, 57]. We also identified novel putative regulatory elements for important cell type-specific regulators, including *Cyp11a1* in steroidogenic progenitors, *Tcf21* in interstitial progenitor cells, and *Wnt6* in pre-Sertoli cells (Supplementary Figure S6). The full list of P2G links in each cluster can be found in (Supplementary Table S3).

Genes often harbor several enhancers in their close proximity, and one or another enhancer or enhancer combination can be used to ensure a proper spatiotemporal expression pattern depending on the cell type or developmental stage [16, 20, 32, 48]. The *Wt1* locus shows at least eight P2G links, and both the intensity and apparent combinatorial usage of these elements vary depending on the cell type (Fig. 4c). Genome browser tracks depicted a combination of predicted enhancers in C3 that were preferentially accessible in CE and supporting cell progenitors/granulosa cells but not detected in pre-Sertoli cells, while C5 putative enhancers were more accessible in pre-Sertoli and pre-granulosa cells. We also observed distal elements that were differentially co-accessible within the *Nr5a1* locus between pre-Sertoli/pre-granulosa cells and steroidogenic progenitors. Careful examination of *Nr5a1* locus revealed steroidogenic progenitor specific peaks (C5 P2G) linked to *Nr5a1*, which overlapped with the previously reported CR8-CR10 fetal Leydig cell enhancer (FLE) (Fig. 4c) [57]. C6 P2G links of *Nr5a1* were more accessible in pre-Sertoli/pre-granulosa cells, suggesting possible cell type-specific enhancer usage.

It is challenging to identify the specific TF binding to the genome and driving gene expression based only on motif enrichment analysis, as TFs from the same family often share a similar motif. Taking the advantage of the integrated data, we identified putative positive TF regulators determined from the correlation between gene expression and the chromVAR motif activity score across single cells, reasoning that the active TF (high motif activity score) should also be actively expressed to maintain its abundance (high mRNA level). Clustering analysis of positive regulators confirmed that the motif binding activity of the identified TFs across cell types closely mirrored their respective gene expression. Reassuringly, this approach also accurately identified known cell type-specific regulators, such as *Sox9* and *Dmrt1* in the pre-Sertoli cluster, *Tcf21* in interstitial/stroma clusters, *Gata4* in pre-granulosa and pre-Sertoli cells, and *Nr5a1* in steroidogenic cells (Fig. 4d). Furthermore, it identified novel TF candidates, such as *Tead4* and *Lmx1a* in CE, *Lef1* in CE/granulosa cells, and *Ets1*/*Fli1* in interstitial/stromal progenitors (Fig. 4e).

Taken together, single-cell multimodal analysis shed new light on the underlying cis-regulatory DNA interactions and the employment of positive TF regulators during gonadal somatic cell type development.

### Chromatin accessibility landscape associated with the male supporting progenitor lineage

**Fig. 5:**
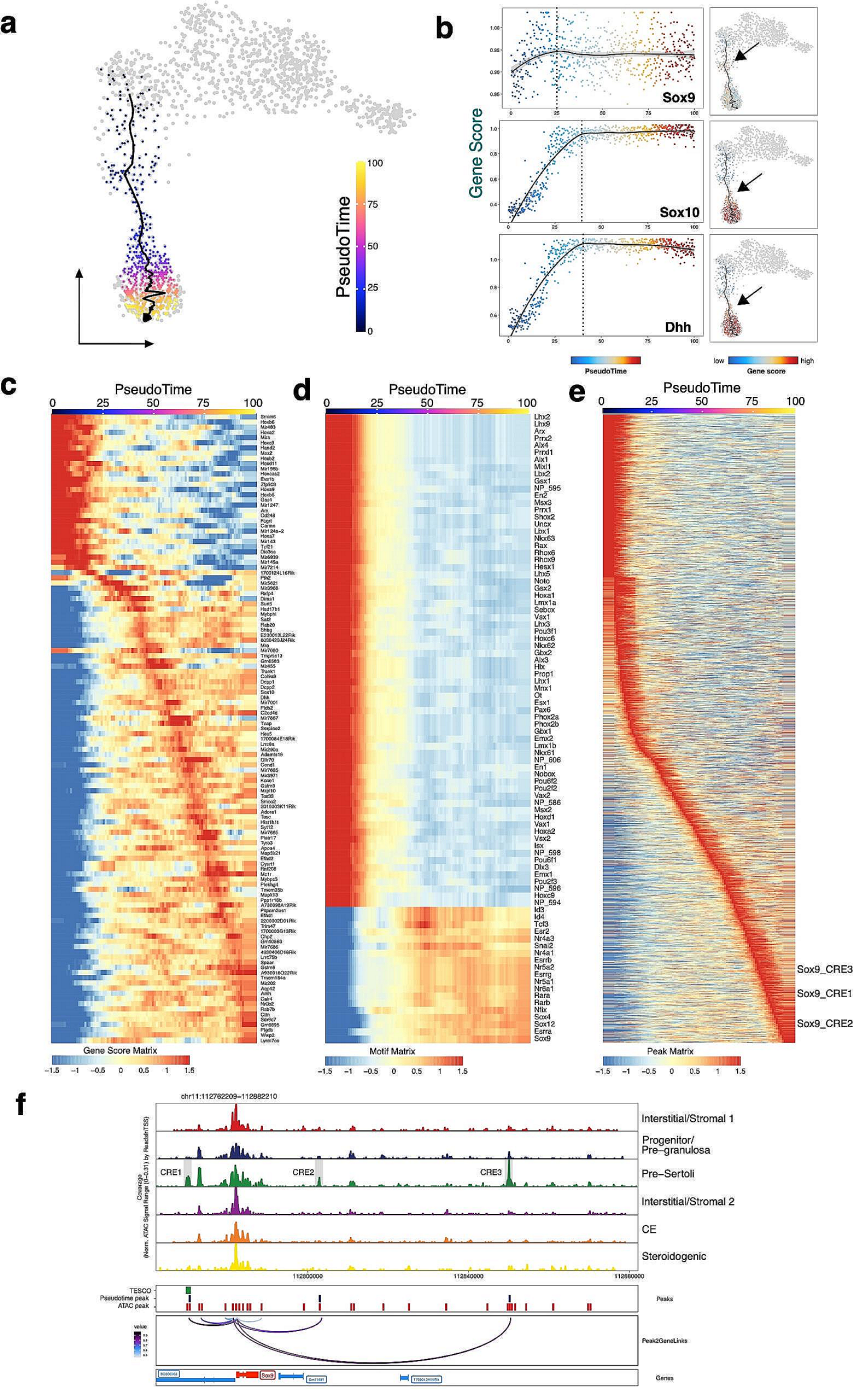
Chromatin accessibility landscape associated with male supporting progenitor lineage. (**a**) scATAC-seq profiles are ordered by pseudotime, corresponding to the Sertoli cell differentiation trajectory. (**b**) Gene scores of selected genes ordered by pseudotime. The dashed lines and arrows point to the location along pseudotime where the highest gene scores are observed. (**c**) Smoothened heatmap showing dynamic gene score of indicated genes along pseudotime. (**d**) Smoothened heatmap showing dynamic motif accessibility of indicated transcription factors along pseudotime. The heatmap was generated using plotTrajectoryHeatmap() function with a varCutOff threshold of 0.9. (**e**) Smoothened heatmap showing peaks with differential accessibility along pseudotime. The peaks labeled with Sox9_CRE1 to 3 correspond to the cis-regulatory element (CRE) regions in Fig. 5e. (**f**) Aggregated scATAC-seq profiles showing peak-to-gene links to the *Sox9* locus overlapped with TESCO and the three potential CREs (CRE1 to 3)

We next examined the early development of Sertoli cells in more detail, because of the more profound chromatin changes in the male supporting cell lineage. We reconstructed the developmental trajectories by ordering the XY cells according to their identity and time points and then generated a pseudotime trajectory (Fig. 5a). The developmental trajectory started from the E11.5 *Wt1* + early progenitor cells (SC5), passed through E11.5 XY cells (SC2), and transitioned to presumptive pre-Sertoli cells (SC3, E11.5–E14.5). We observed that several Sertoli cell-associated genes changed chromatin accessibility (gene score) along the pseudotime, such as *Sox10* and *Dhh* (Fig. 5b and c). *Sox10* began to be expressed in pre-Sertoli cells shortly after *Sox9* upregulation and *Sox10* gain-of-function causes XX sex reversal [51]. *Dhh* initiated expression shortly after the activation of *Sry* in pre-Sertoli cells and is one of the earliest indications of male sexual differentiation [7]. Consistently, both genes exhibited peak accessibility in the middle of the trajectory, noticeably occurring later than *Sox9*, further highlighting the robustness of pseudotime analysis (Fig. 5b). Interestingly, the scATAC-seq data revealed that numerous miRNA loci are associated with dynamic chromatin accessibility along the trajectory. Since differences in open chromatin correlate with changes in miRNA expression levels [66], these results hinted at miRNA candidates with potential roles in Sertoli cell differentiation, which complements standard single-cell RNA-seq techniques that do not capture miRNAs due to the lack of a polyA tail (Fig. 5c).

We further identified TFs with changes in motif accessibility along the trajectory (Fig. 5d). The motif accessibility landscape can be briefly divided into three stages. Before Sertoli cell specification, the CE-enriched transcription factors, including *Arx* and *Lhx9*, showed higher motif accessibility. The motif of homeobox protein *Emx2*, which is essential for the formation of genital ridge, is also more enriched [34]. In addition, other homeobox genes, such as *Hoxa1*, *Hoxc6* and *Hoxd1*, are more active before Sertoli cell specification. During the transitional stage, there is an enrichment of *Id3* and *Id4* motifs. The Drosophila homolog of the ID proteins, extramacrochaetae (emc), is involved in sex determination [59]. *Id3* and *Id4* may have similar regulatory roles in the development of mouse gonads. Moreover, *Tcf3*, *Esr2*, and *Snai2* also showed an increase of motif activity in the middle of the trajectory, suggesting that this may contribute to Sertoli cell fate specification. In accordance with this observation, *Snai2* mutant mice displayed gonadal defects [50]. At late stage, the retinoic acid (RA) receptors *Rara* and *Rarb* showed increased motif accessibility, consistent with RA’s role to induce Sertoli cell proliferation [47]. *Sox9* also showed increased motif activity at late stage, concordant with the role of SOX9 in the maintenance of the Sertoli cell fate [4]. We further explored the regulation of the differentiation process by correlating ChromVAR TF motif activity with integrated TF mRNA expression along this pseudotime trajectory. This analysis allowed us to prioritize positive regulators of the differentiation process beyond TF motif activity alone (Supplementary Figure S7).

We then tracked the individual peaks that showed accessibility changes along the pseudotime of Sertoli cell differentiation (correlation > 0.95) (Fig. 5e, Supplementary Table S5). During male sex determination, SRY activates male-specific transcription of *Sox9* and the *Sox9* mRNA expression becomes detectable in XY pre-Sertoli cells at E11.5 [44, 55]. We readily recovered a region 13 kb upstream of the *Sox9* TSS change along the pseudotime, which overlapped with the testis-specific enhancer of *Sox9* (TES), including the core element of TES, TESCO (Fig. 5e). Previous research revealed that the TES accounted for only approximately half of the normal levels of *Sox9* expression in the mouse testis, and that one or more other *Sox9* enhancers remain to be discovered [22]. We identified two additional regions located 21 kb 3’ and 68 kb 3’ of *Sox9*. Importantly, these two regions were identified as peaks linking *Sox9* mRNA expression in the P2G analysis (Fig. 5f), which represent potential regulatory elements involved in both *Sox9* gene regulation and Sertoli cell differentiation. Furthermore, we focused on the 682 distal intergenic regions to better understand the cis-regulatory mechanisms that underlie gene expression dynamics (Supplementary Table S4). ToppGene suite analysis of the associated genes revealed that they were enriched in the Notch pathway (e.g., *Yy1* and *Notch1*) and the Hippo pathway (e.g., *Smad3* and *Smad7*) (Supplementary Figure S8).

Taken together, we have demonstrated that the development of Sertoli cells involves drastic and distinct chromatin remodeling together with unique TF regulation.

### TF candidates in early lineage specification of steroidogenic populations

**Fig. 6:**
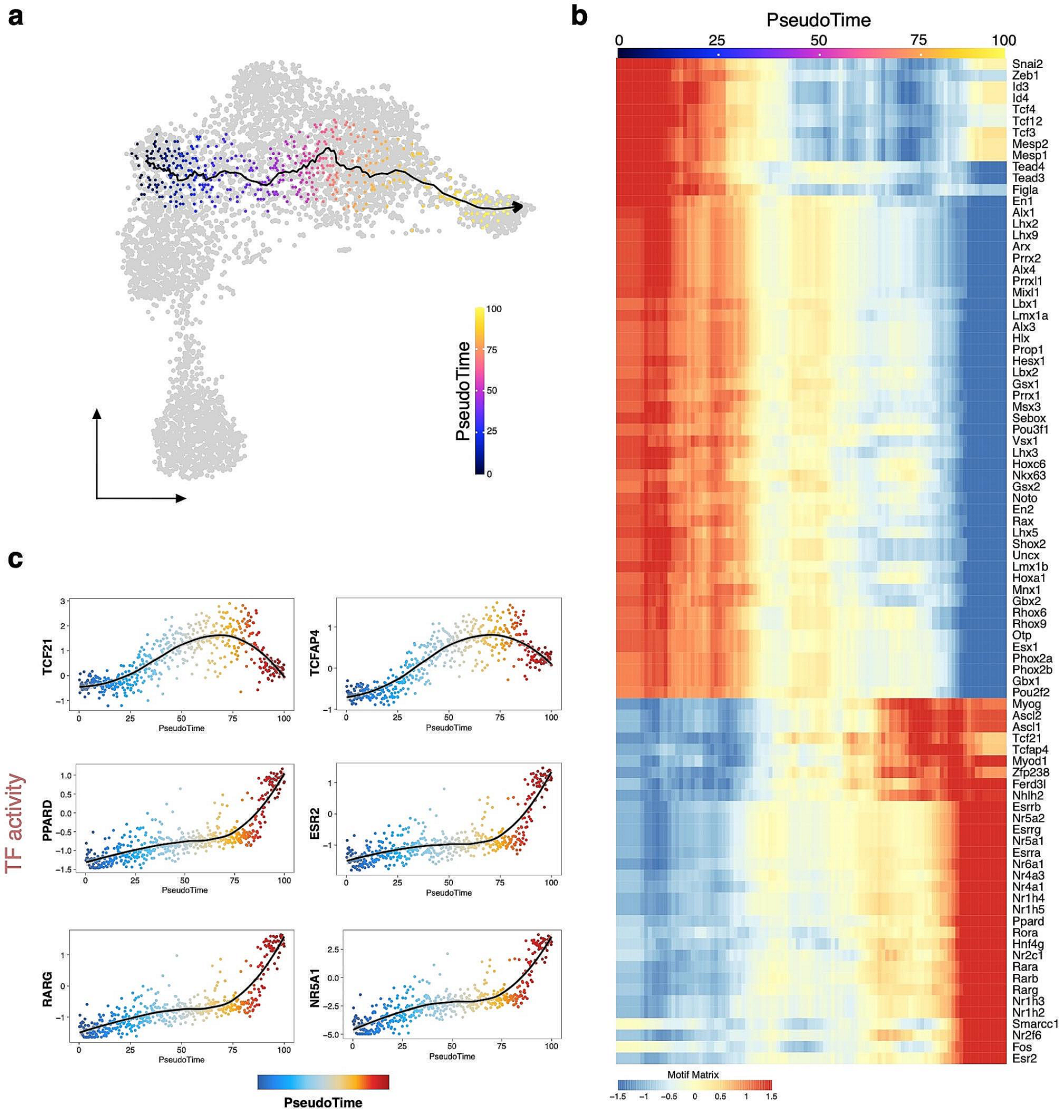
Transcription factor candidates in early lineage specification of steroidogenic populations. (**a**) scATAC-seq profiles are ordered by pseudotime, corresponding to the steroidogenic lineage specification trajectory. (**b**) Smoothened heatmap showing dynamic motif accessibility of indicated transcription factors (TFs) along pseudotime. (**c**) Motif accessibility of selected TFs ordered by pseudotime

The steroidogenic progenitor cluster (SC6) emerged at E12.5, together with the interstitial somatic cell cluster (SC1) (Fig. 3b). The mix of both XX and XY cells in these clusters indicates that the chromatin accessibility landscapes of XY interstitial and XX stromal progenitors are similar and the resemblance is maintained after both XY and XX progenitor cells acquire a steroidogenic precursor fate (Fig. 3b). Steroidogenic progenitors can originate from the cells in the interstitial compartment derived from the coelomic epithelium, as well as from the mesonephros [1, 15, 17]. To explore the lineage specification of steroidogenic cells, we conducted pseudotime analysis focusing on SC5, SC1, and SC6 at E12.5, corresponding to the progression from the CE to interstitial cells and finally to steroidogenic progenitors (Fig. 6a). We observed elevated *Tcf21* TF motif activity when cells transited to SC6, suggesting *Tcf21*’s role in interstitial/stromal progenitor specification (Fig. 6b and c). Various studies have shown TCF21’s role in male sex determination and testis somatic cell differentiation [6, 13]. We identified higher activity of *Nr5a1* in the late stage of the trajectory, consistent with its role in steroidogenic cell differentiation [35]. Novel candidates include *Tcfap4* and the retinoic acid receptors (*Rara*, *Rarb* and *Rarg*), while the nuclear estrogen receptor *Esr2* and Peroxisome Proliferator-activated Receptor-D (*Ppard*) also merit further investigation (Fig. 6b and c). Through our analysis of positive regulators along pseudotime, we were able to further prioritize potential candidates, including *Meis3* and *Nr3c1*, as potential drivers of the differentiation process (Supplementary Figure S9).

### Distinct TF dynamics and chromatin regulation during male and female germ cell development

**Fig. 7:**
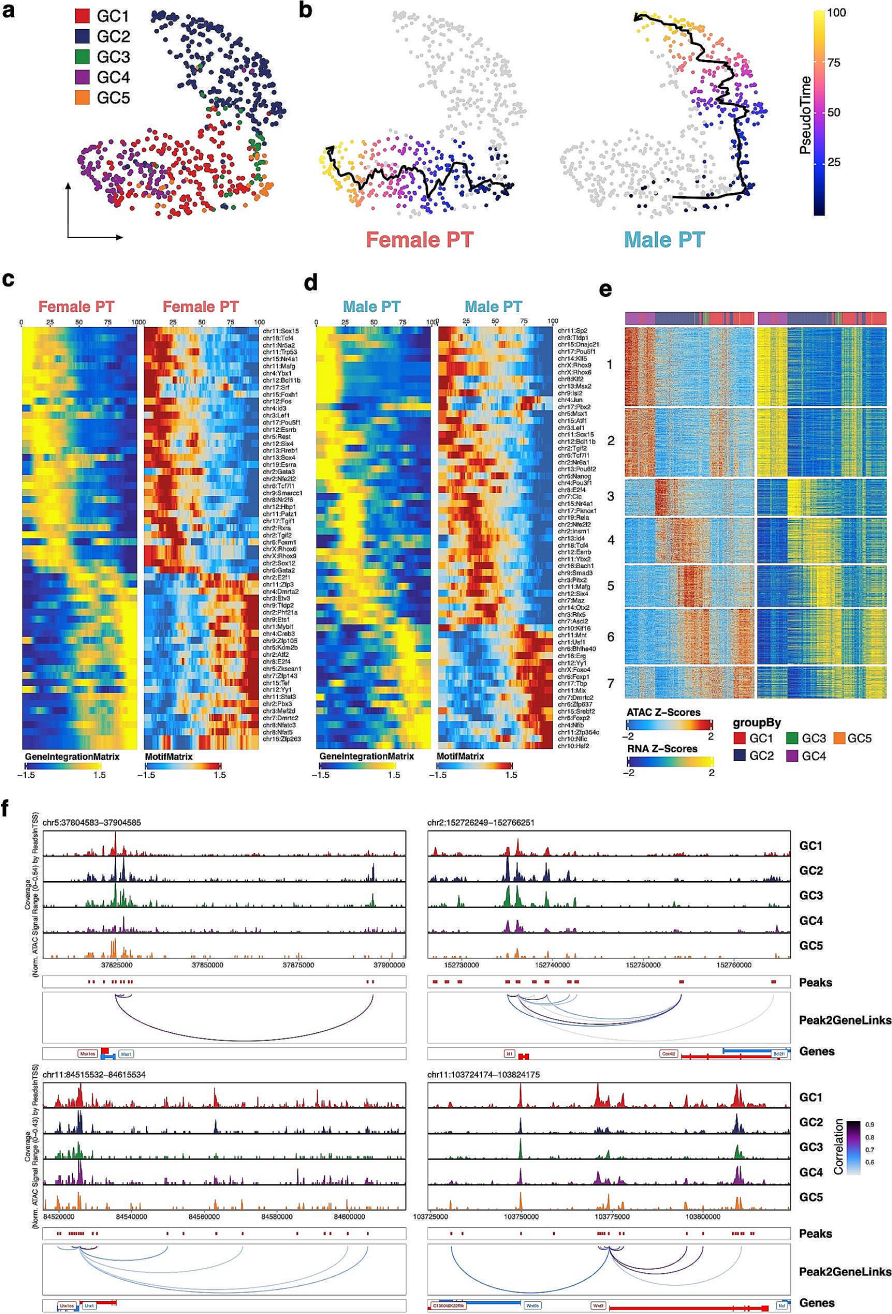
Transient patterns of transcription factor expression and regulatory elements during germ cell sex specification. (**a**) UMAP representation of all female and male germ cells. Cells are colored by clustering based on integration with scRNA-seq data. (**b**) scATAC-seq profiles are ordered by pseudotime, corresponding to the female (left) and male (right) germ cell development trajectory. (**c**) Heatmap showing dynamic gene expression (left) and transcription factor (TF) motif activity (right) of indicated TFs along pseudotime for gene-motif pairs of the female germ cell trajectory. (**d**) Smoothened heatmap showing dynamic gene expression (left) and TF motif accessibility (right) of indicated TFs along pseudotime for gene-motif pairs of the male germ cell trajectory. (**e**) Heatmaps showing TF motif activity (left) and gene expression (right) of 21,710 peak-to-gene links across germ cell clusters (corCutOff = 0.6). (**f**) Aggregated scATAC-seq profiles showing peak-to-gene links to the *Msx1* (upper left), *Id1* (upper right), *Lhx1* (lower left) and *Wnt3* (lower right) loci

We then investigated the development of the female and male germ cells. The germ cells from E11.5 to E14.5 were re-clustered into five cell clusters, namely GC1 to GC5 (Fig. 7a, Supplementary Figure S10a). The cells were branched from GC5, indicating that sex specification at the chromatin level of germ cells begins at around E11.5 and E12.5. By inferring the chrY scores, GC2 and GC3 were the male germ cells while GC1 and GC4 were the female germ cells (Supplementary Figure S10b). We then reconstructed the pseudotime developmental trajectories by ordering the female and male clusters following developmental time points (Fig. 7b). We also integrated our scATAC-seq data with previous scRNA-seq data [41]. To determine the TFs driving germ cell development in males and females, we first identified a list of genes with dynamic expression and a list of TF motifs with differential binding activity throughout the two trajectories. We then correlated the gene expression (inferred by scRNA-seq) and TF binding activity (chromaVAR score inferred by scATAC-seq) to identify a list of high-confidence TFs with dynamic regulation along the trajectories (Fig. 7c and d).

Comparing the heatmap side-by-side reveals a distinct pattern of TF reconfiguration in female and male germ cell differentiation. In females, there is a two-step process involving the down-regulation of pluripotency markers (*Oct4* and *Lef1*) among other TFs (e.g., *Rhox6* and *Rhox9*), followed by the upregulation of another set of TFs, including *Yy1* and *Stat3*. *Stat3* has been shown to be differentially distributed in the cytoplasm of mature oocytes [46]. In contrast, male germ cell differentiation appears to involve a more complex, multi-step process. Male germ cell differentiation also involved down-regulation of pluripotency markers (*Oct4* and *Lef1*) but followed by a transient activation of TFs including *Id4* and *Six4*, before later stage upregulation of TFs such as *Yy1* and *Foxo4*. *Foxo4* is essential for spermatogonial stem cell self-renewal [21].

Utilizing the aforementioned analytical framework, we identified the P2G links of germ cells (Supplementary Table S5). A total of 23,004 germ cell-specific P2G links were clustered into seven clusters and GO analysis was performed (Fig. 7e, Supplementary Figure S10c). As expected, the GO terms related to meiosis were enriched in the genes of the P2G links of Clusters 1 and 2, which were mainly upregulated in E14.5 female germ cells. For the male germ cells, the GO terms associated with the P2G links of Clusters 3 to 6 included RNA processing, cell cycle and ribosome biogenesis, which is consistent with the fact that male germ cells are undergoing active cell division during this period. Our analysis further revealed putative regulatory elements specific to male (*Msx1* and *Id1*) and female (*Lhx1* and *Wnt3*) germ cells (Fig. 7f).

## Discussion

In addition to the transcriptome, the epigenome is another element crucial to understanding the mechanisms underlying many developmental processes, including gonad development [29]. To date, our knowledge about the epigenetic regulation of gonadal development at the single-cell level is still limited, especially the intersection between transcriptome and chromatin accessibility. In this study, we conducted an in-depth analysis of the temporal dynamics of the chromatin accessibility landscape at single cell resolution for mouse embryonic gonads.

scATAC-seq analysis offers several insights into the epigenomic states underlying cellular transition during gonadogenesis. First, chromatin accessibility landscapes of XX and XY supporting cell progenitors prior sex differentiation at E11.5 are similar. Hence, the specification of supporting cell lineages is independent of sex. Secondly, the chromatin reconfiguration during Sertoli and granulosa cell lineage specification shows a temporal asymmetry since pre-Sertoli cells have already appeared in E11.5 bipotent gonads. Third, the shared chromatin accessibility landscape between the pre-granulosa cell state (E12.5 to E14.5) and the progenitor state (E11.5), as opposed to Sertoli cells, suggesting the supporting cell progenitors exhibit a bias toward a female chromatin state before subsequently being diverted in XX or XY gonads. Fourth, both sexes have similar patterns of chromatin accessibility during interstitial or stromal lineage commitment and differentiation.

Low-abundance essential genes, including many TFs, are particularly affected by the limitations of scRNA-seq such as dropouts (false zero expression estimates) and high variability [36]. This means that the biological information conveyed by lowly expressed TFs is lost. scATAC-seq allows the detection of TF activity in single cells, which significantly increases the ability to search for transcriptional regulators. Through integrated analysis of both chromatin accessibility and gene expression, our study provided a holistic view of temporal TF regulation during Sertoli and steroidogenic cell differentiation, which serves as a fundamental base to reveal the mechanisms involved in gonadogenesis. Examples include *Tcf3*, *Esr2* and *Snai2* for Sertoli differentiation, and *Tcf21* and *Tcfap4 for* steroidogenic differentiation. Hopefully, these TF candidates will be useful for improving the in vitro induced system for Sertoli and steroidogenic cells, which may provide potential treatments for male infertility in the future.

Integrating previous work profiling the transcriptome of gonad development, our multiomics analysis not only allowed us to prioritize TFs that are both expressed and active but also to identify the probable CREs of critical cell type-specific genes based on the P2G linkages. More importantly, our study highlights the benefit of scATAC-seq analysis in identifying cell type-specific enhancers, exemplified by the re-discovery of the Enh13 enhancer of *Sox9*. ​​Complex spatially and temporally defined expression patterns of gonadal genes have been observed in development. For instance, *Nr5a1* expression starts at the progenitor cell stage and reaches a plateau in matured steroidogenic cells and supporting cells. *Wt1* is also expressed in different gonadal cell types including the CE, supporting cell progenitors, Sertoli cells and granulosa cells [49, 54]. The multiple cell type-specific CREs of *Nr5a1* and *Wt1* that have been identified may be responsible for this complex expression pattern, each acting independently and controlling transcription in different cell types at different developmental stages. Researchers interested in how gene regulation is mediated in the developing gonad can benefit immediately from the identification and categorization of these candidate CREs across cell types.

Taken together, our findings establish a groundwork for future clarification of mechanisms in mammalian gonad development, as well as a better knowledge of the genetic basis of human sex development disorders and infertility.

### Limitations of the study

The relatively small number of cells included in our study limited our ability to explore only major cell types in the gonad. To capture the full complexity of gonad development, larger-scale studies or pre-enrichment methods followed by scATAC-seq are necessary. The mix of male and female gonads during sampling, along with homologous genes on X and Y chromosomes and potential alignment errors, introduces challenges in accurately assigning sex to cells based on sex chromosome reads bioinformatically. As a result, while we have explored certain aspects of sex-specific processes, a complete understanding of the intricacies of sex determination requires further investigation. Our study focuses on chromatin accessibility, one of the many attributes of chromatin status. We expect that the advent of new single-cell sequencing methods for the study of DNA and histone modification, combined with single-cell transcriptome/chromatin accessibility data, will be instrumental in advancing our understanding of the epigenetic regulation at play in each somatic cell lineage during mammalian gonad development. Performing scRNA-seq and scATAC-seq in the same cells will further enhance the accuracy of linking the chromatin landscape with gene expression. Furthermore, analysis of human gonad development using the same framework is critical to validate our results on mice. Finally, we recognize that further validation and functional studies will be necessary to confirm the regulatory roles of the identified TFs and cis-regulatory elements. We believe that these limitations provide opportunities for future research to build upon the findings of our study and to further enhance our understanding of the complex regulatory networks that govern gonad development.

## Methods

### scATAC-seq analysis

#### Sample collection

Mouse strain C57BL/6J was purchased from the Jackson Laboratory. Pregnant females were euthanized by cervical dislocation at embryonic days: E11.5, E12.5, E13.5 and E14.5, which the day with the presence of vaginal plugs was defined as E0.5. For the embryos at E11.5, whole genital ridges (including gonad and mesonephros) were dissected. For the embryos at E12.5, E13.5 and E14.5, gonads were dissected free of the mesonephros. The sexing step was omitted to reduce the sample processing time, and the gonads from both sexes were pooled in equal numbers for subsequent steps. The XX and XY gonads at the same time point were pooled together. Each time point contained at least 6 embryos.

#### Cell lysis and tagmentation

Cell tagmentation was performed according to SureCell ATAC-Seq Library Prep Kit (17,004,620, Bio-Rad) User Guide (10,000,106,678, Bio-Rad) and the protocol based on Omni-ATAC was followed [10]. In brief, washed and pelleted cells were lysed with the Omni-ATAC lysis buffer containing 0.1% NP-40, 0.1% Tween-20, 0.01% digitonin, 10 mM NaCl, 3 mM MgCl2 and 10 mM Tris-HCl pH 7.4 for 3 min on ice. The lysis buffer was diluted with ATAC-Tween buffer that contains 0.1% Tween-20. Nuclei were counted and examined under microscope to ensure successful isolation. Nuclei were resuspended in tagmentation mix, buffered with 1×PBS supplemented with 0.1% BSA and agitated on a ThermoMixer for 30 min at 37 °C. Equal number of nuclei and equal ratio of cells/Tn5 transposase were used to minimize potential batch effect. Tagmented nuclei were kept on ice before encapsulation.

#### scATAC-seq library preparation and sequencing

Tagmented nuclei were loaded onto a ddSEQ Single-Cell Isolator (Bio-Rad). scATAC-seq libraries were prepared using the SureCell ATAC-Seq Library Prep Kit (17,004,620, Bio-Rad) and SureCell ATAC-Seq Index Kit (12,009,360, Bio-Rad). Bead barcoding and sample indexing were performed with PCR amplification as follows: 37 °C for 30 min, 85 °C for 10 min, 72 °C for 5 min, 98 °C for 30 s, eight cycles of 98 °C for 10 s, 55 °C for 30 s and 72 °C for 60 s, and a single 72 °C extension for 5 min to finish. Emulsions were disrupted and products were cleaned up using Ampure XP beads. Barcoded amplicons were further amplified for 8 cycles. PCR products were purified using Ampure XP beads and quantified on an Agilent Bioanalyzer (G2939BA, Agilent) using the High-Sensitivity DNA kit (5067 − 4626, Agilent). Libraries were sequenced on HiSeq 2000 with 150 bp paired-end reads.

#### Sequencing reads preprocessing

The Bio-Rad ATAC-seq Analysis Toolkit was used to process the sequencing data. This streamlined computational pipeline includes tools for FASTQ debarcoding, read trimming, alignment, bead filtration, bead deconvolution, cell filtration, and peak calling. The reference index was built upon the mouse genome mm10. First, barcodes were parsed out of the R1 reads of the FASTQ files and appended to the read name for all reads with valid barcodes. Reads failed debarcoding were discarded. The alignment step aligned debarcoded reads to the reference genome using BWA (http://bio-bwa.sourceforge.net/). Reads with low mapping quality, those not properly paired, and those mapped to the mitochondrial genome were removed. The BioRad SureCell scATAC-seq kit used in this study allows multiple beads per droplet, necessitating barcode merging. Bead-based ATAC-seq processing (BAP) was employed (https://github.com/caleblareau/bap) to identify systematic biases (i.e., reads aligning to an inordinately large number of barcodes), deduplicate reads and perform merging of multiple bead barcode instances associated with the same cell (“bead deconvolution”). For generation of the fragments file, which contain the start and end genomic coordinates of all aligned sequenced fragments, sorted bam files were further processed with “bap-frag” module of BAP. Downstream analysis was performed with SnapATAC [19] and ArchR [24]. Sorted bam file of each sample was structured into hdf5 snapfiles (single-nucleus accessibility profiles) using snaptools version 1.4.1. Cell-by-bin matrices were created with a bin size of 5,000 bp. SnapATAC package was used to merge snapfiles based on the same bin coordinates. Peak coordinates obtained from Bio-Rad ATAC-seq Analysis Toolkit were loaded on snapfiles to generate a cell-by-peak matrix. Fragment files were used to create the Arrow file in the ArchR package.

#### Clustering analysis

We filtered out low-quality nuclei with stringent selection criteria, including read depth per cell (> 2,000) and TSS enrichment score (> 4). Potential doubles were further removed based on the ArchR method. Bin regions were cleaned by eliminating bins overlapping with ENCODE Blacklist regions, mitochondrial DNA as well as the top 5% of invariant features (house-keeping gene promoters). Dimensionality reduction was performed using a nonlinear diffusion map algorithm available in the SnapATAC 1.0.0 package to produce 50 eigen-vectors of which the first 10 were selected in order to generate K Nearest Neighbor graph with K = 15. The clustering was performed using Leiden Algorithm available in the package leidenalg version 0.7.0 with a resolution of 0.8 and UMAP embedding were generated using umap-learn.

#### Gene score and transcription factor activity estimation

ArchR was used to estimate gene expression for genes and TF motif activity from single cell chromatin accessibility data. Gene scores were calculated using the addGeneScoreMatrix function with gene score models implemented in ArchR. addDeviationsMatrix function was used to compute enrichment of TF activity on a per-cell basis across all motif annotations based on chromVAR (chromVAR_0.3). The chrY scores were calculated based on the number of reads mapped to the Y chromosome, normalized to sequencing depth per 10 K reads. Cells were designated as male if ChrY reads were present (chrY score > 0).

#### Marker gene and DAR analysis

To identify marker genes based on gene scores or marker peaks, we used the getMarkerFeatures function in ArchR with useMatrix = “GeneScoreMatrix” or “PeakMatrix”, respectively. ArchR performed Wilcoxon testing for comparison after accounting for the biases (i.e., “TSSEnrichment” score and “log10(nFrags)”. These p-values are then adjusted for multiple hypothesis using the FDR method. Cell type-specific DARs were visualized as a heatmap using the markerHeatmap() function. Motif enrichment on marker peaks were identified using getMarkerFeatures(). Motif footprinting was generated using the getFootprints() function. DARs were annotated using ChIPseeker and the GO term enrichment of DARs associated genes were performed using clusterProfiler.

#### Integration with scRNA-seq data

The integration of scATAC-seq with published time-series scRNA-seq data of all gonadal cells (GEO: GSE184708), somatic cell subsets in the gonads (DNA Data Bank of Japan: DRA011172) and germ cells (GEO: GSE136220) were carried out using ArchR [41, 58]. A new GeneIntegrationMatrix corresponding to the predicted linked gene expression from scRNA-seq was added using addGeneIntegrationMatrix(). Putative cis-regulatory elements were identified based on correlated peak accessibility and linked gene expression (peak-to-gene linkages) across different cell types using addPeak2GeneLinks().

#### Trajectory analysis

Trajectory analysis was performed in ArchR. addTrajectory() function in ArchR was used to construct trajectory on the UMAP embedding. Pseudo-time versus the gene score, peak accessibility and Motif activity were visualized using plotTrajectory() or plotTrajectoryHeatmap() function. To identify positive TF regulators by integration of gene scores with motif accessibility across pseudo-time, we used the correlateTrajectories() function which takes two SummarizedExperiment objects retrieved from the getTrajectories() function.

### Statistical analysis

Assessment of statistical significance was performed using two-tailed unpaired t-tests, one-way ANOVA with Tukey multiple comparisons tests or Chi-squared tests. Statistical analysis was performed using GraphPad Prism v8. Associated P values are indicated as follows: *P < 0.05; **P < 0.01; ***P < 0.001; ****P < 0.0001; not significant (ns) *P* > 0.05.

### Electronic supplementary material

Below is the link to the electronic supplementary material.

Supplementary Material 1

Supplementary Material 2

Supplementary Material 3

Supplementary Material 4

Supplementary Material 5

Supplementary Material 6

## Data Availability

Raw data, ATAC-seq fragment files, and associated metadata from this study have been deposited in the NCBI gene expression omnibus (GEO) under accession number GSE218995. Code for producing the majority of analysis from this paper is available on GitHub at https://github.com/liaojinyue/mouse-gonad-scATAC-seq.
