# Supplementary material for: The single-cell chromatin landscape in gonadal cell lineage specification
Source: BMC Genomics. 2024 May 13;25:464. doi: 10.1186/s12864-024-10376-1 (PMC11092170; doi:10.1186/s12864-024-10376-1)
Supplement: Supplementary file 1 — Supplementary Material 1 [file 12864_2024_10376_MOESM1_ESM.docx]

**Supplementary Figures and Legends**

**
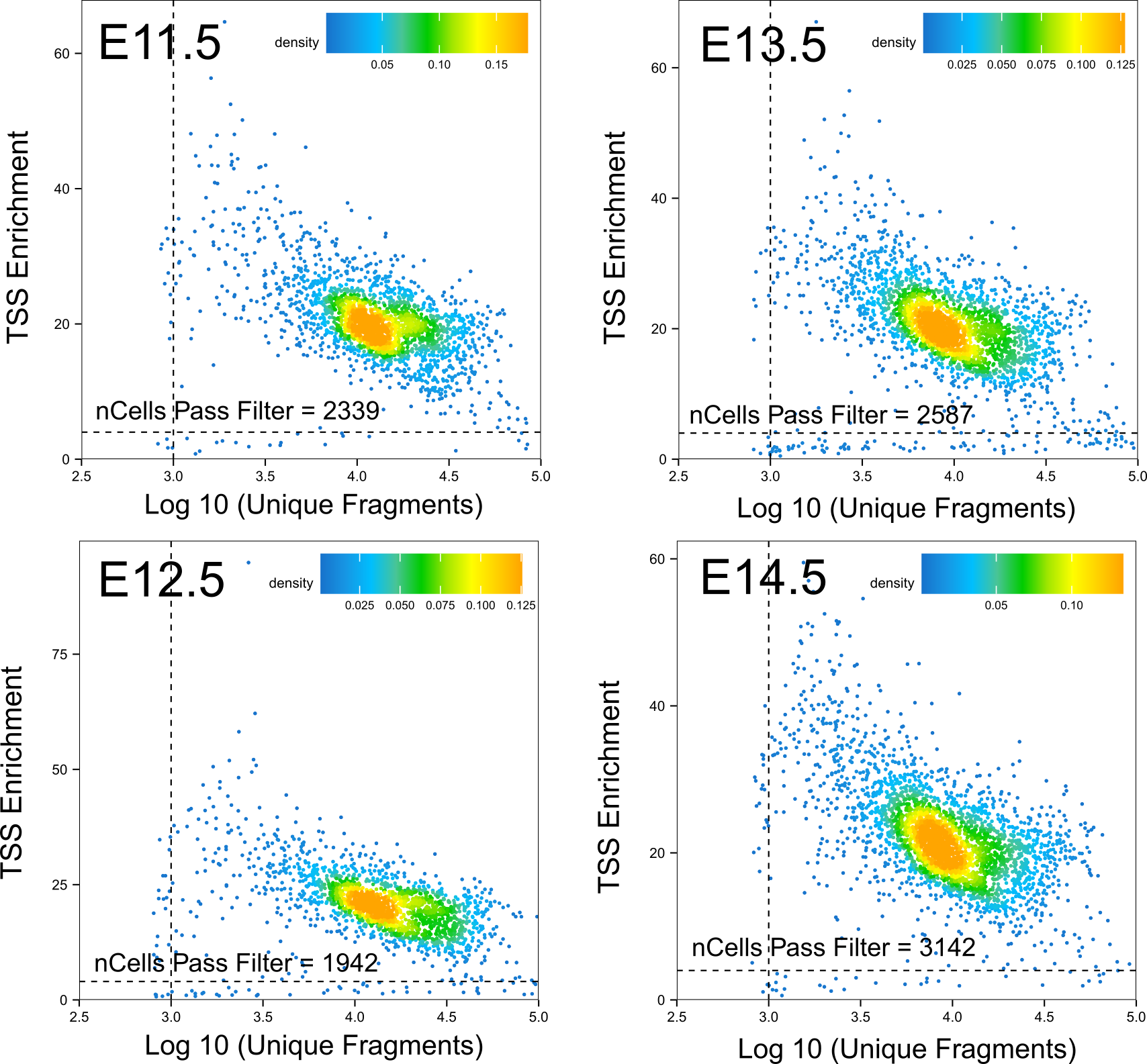
**

**Supplementary Figure S1. Quality assessment metrics for scATAC-Seq libraries, related to Figure 1.**

Plot of TSS enrichment score versus the total number of unique fragments of each sample. Only cells lying in the upper right quadrant (marked by dashed lines) are retained for downstream analysis.


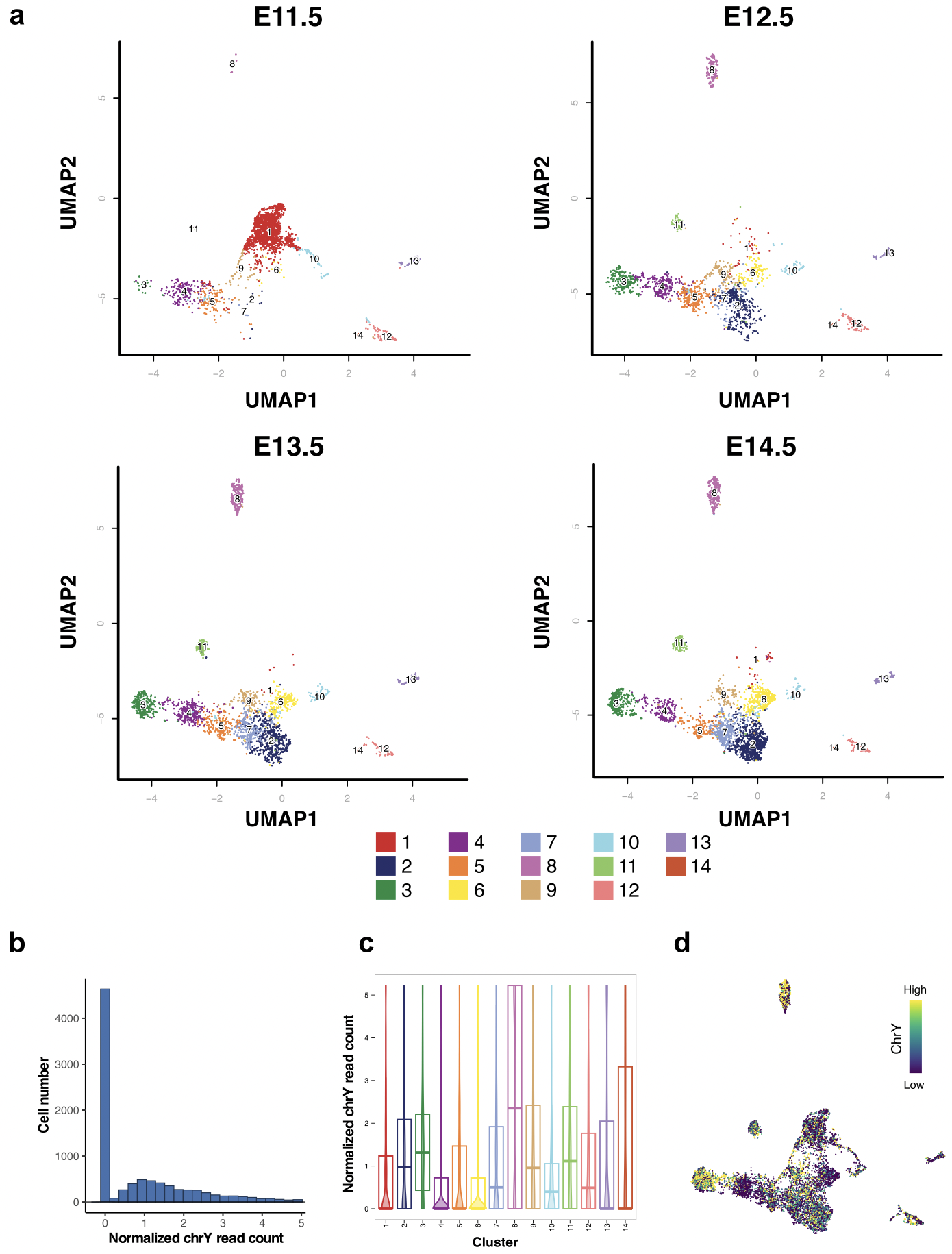


**Supplementary Figure S2. Distribution of all cells according to time points and cell sex assignment, related to Figure 1.**

**a**) UMAP representation of all cells among the four time points. b) Histogram showing normalized chrY read count of all cells. c) Violin plot showing normalized chrY read count of each cluster. d) UMAP representation of all cells captured from all four time points colored by chrY score.

**
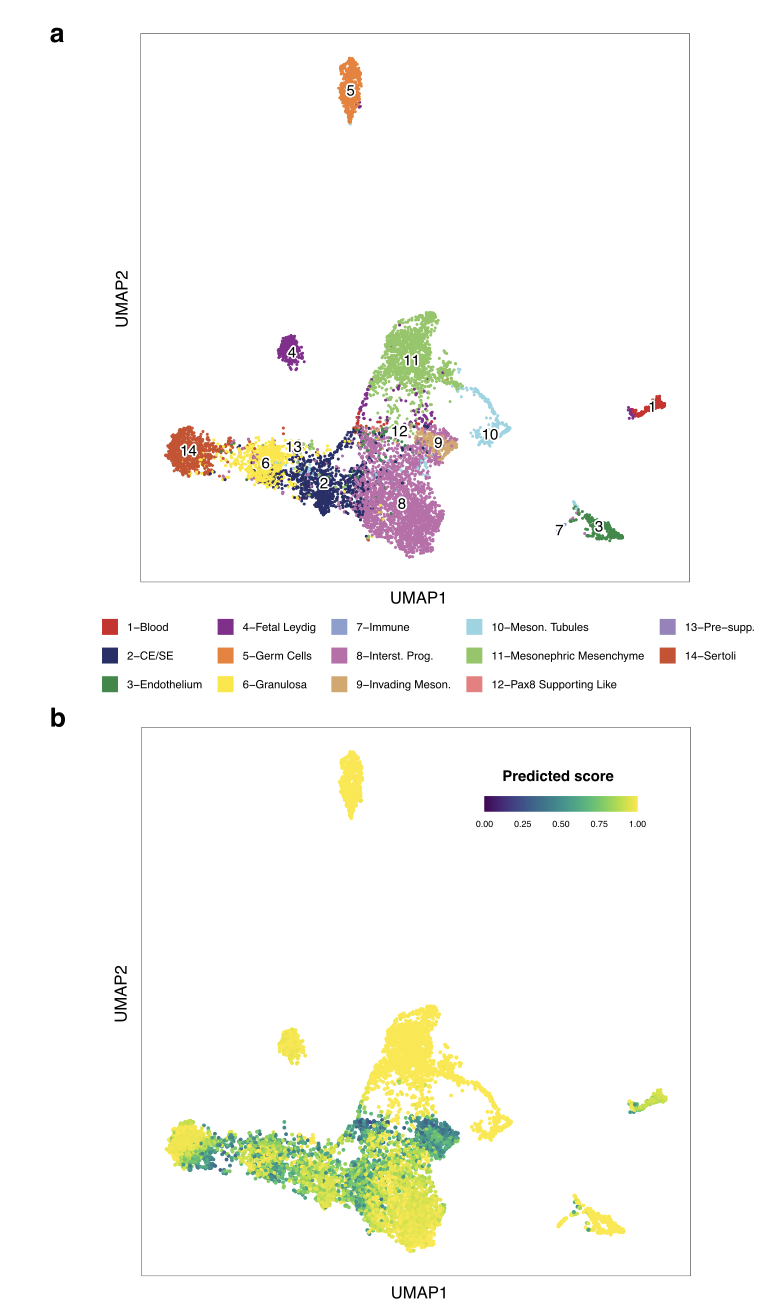
**

**Supplementary Figure S3. Integration of all gonadal cell scRNA-seq data (GSE184708) and scATAC-seq data (this study), related to Figure 1.**

**a)** The UMAP generated from scATAC-seq data was labeled with annotations corresponding to cell types identified from scRNA-seq analysis. CE, coelomic epithelial cells; SE, surface epithelial cells; Meson. Tubules, mesonephric tubules; Pre-Sup., pre-supporting cells; Sertoli, Sertoli cells; Granulosa, pre-granulosa cells; Invading Meson., invading mesonephric cells; **b)** Prediction score of data integration based on ArchR analysis.

**
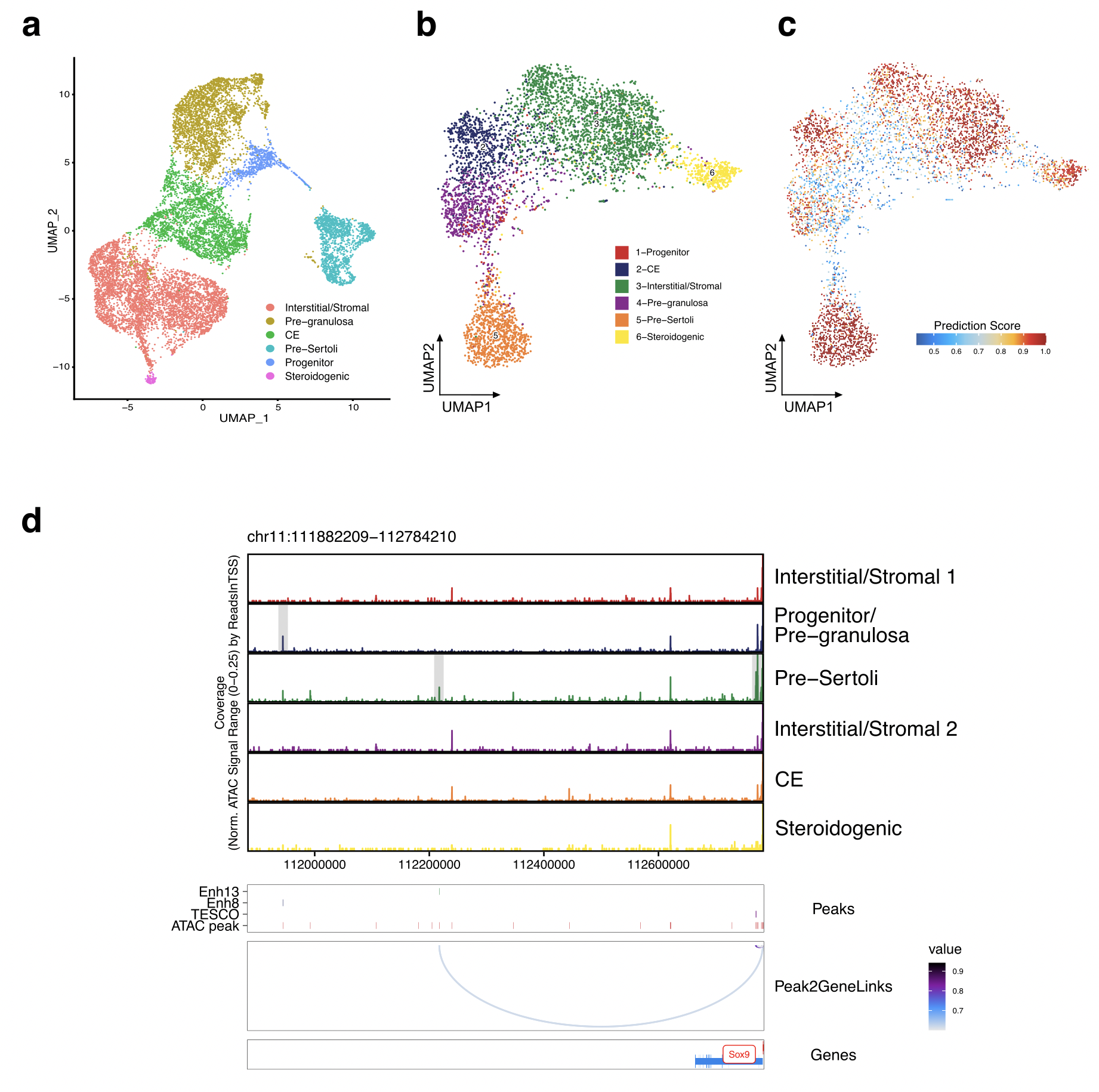
**

**Supplementary Figure S4. Reanalysis and integration of gonadal somatic cell scRNA-seq data, related to Figure 4.**

**a)** UMAP representation of distribution of gonadal somatic cells from published scRNA-seq data. **b)** UMAPs of scATAC-seq data labeled with annotation based on the scRNA-seq cell type. **c)** Prediction score of data integration based on ArchR analysis. **d)** Aggregated scATAC-seq profiles showing peak-to-gene links to *Sox9*, highlighting previously reported Enh13 and TESCO enhancer regions. Noted a higher accessibility to Enh8 region in the pre-granulosa cluster, which is consistent with the previous report.


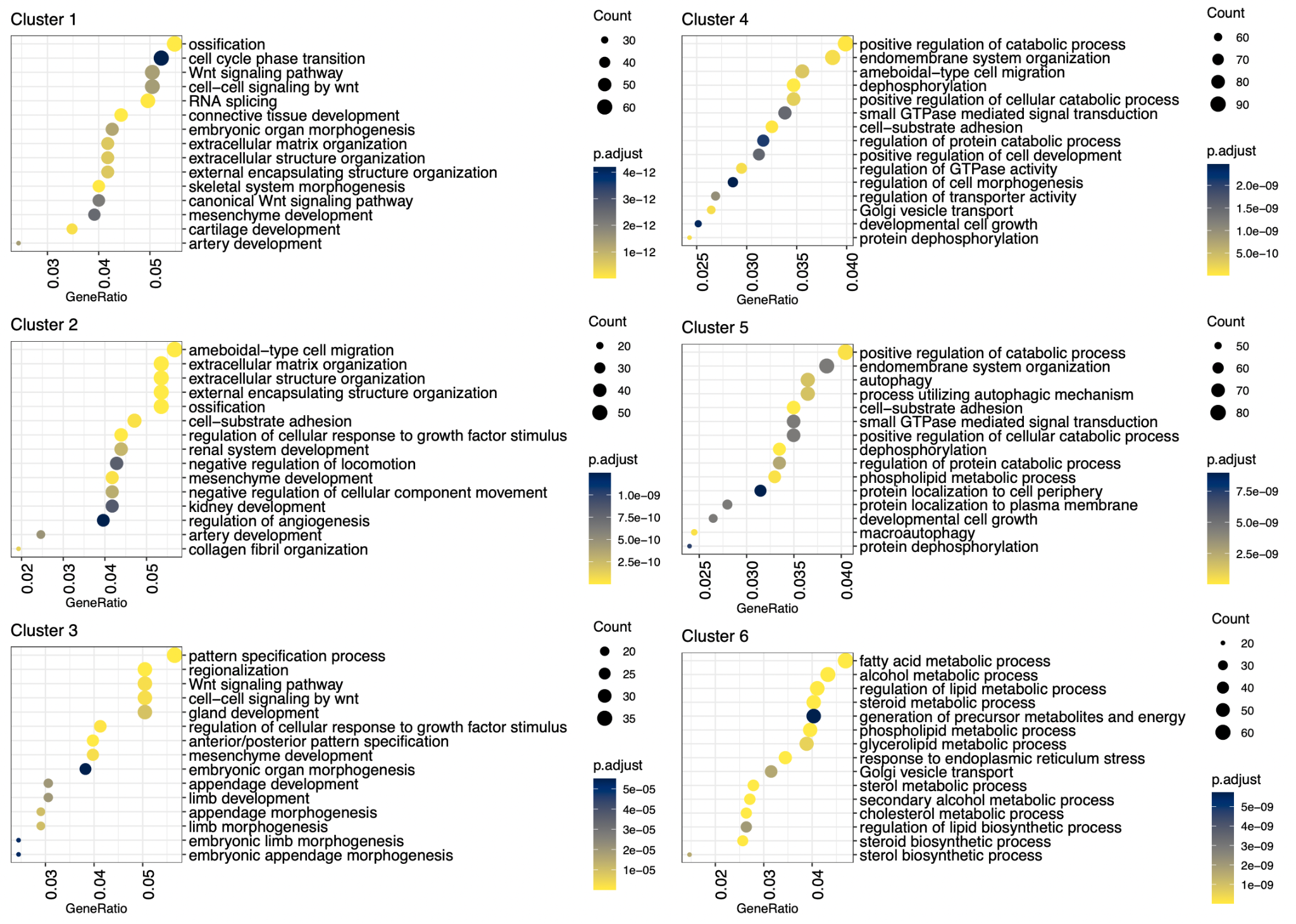


**Supplementary Figure S5. Characterization of peak-to-gene links across somatic cell types, related to Figure 4.**

Top results from the Gene Ontology (GO) enrichment test showing the terms associated with peak-to-gene links in each cluster identified in Figure 4b.

**
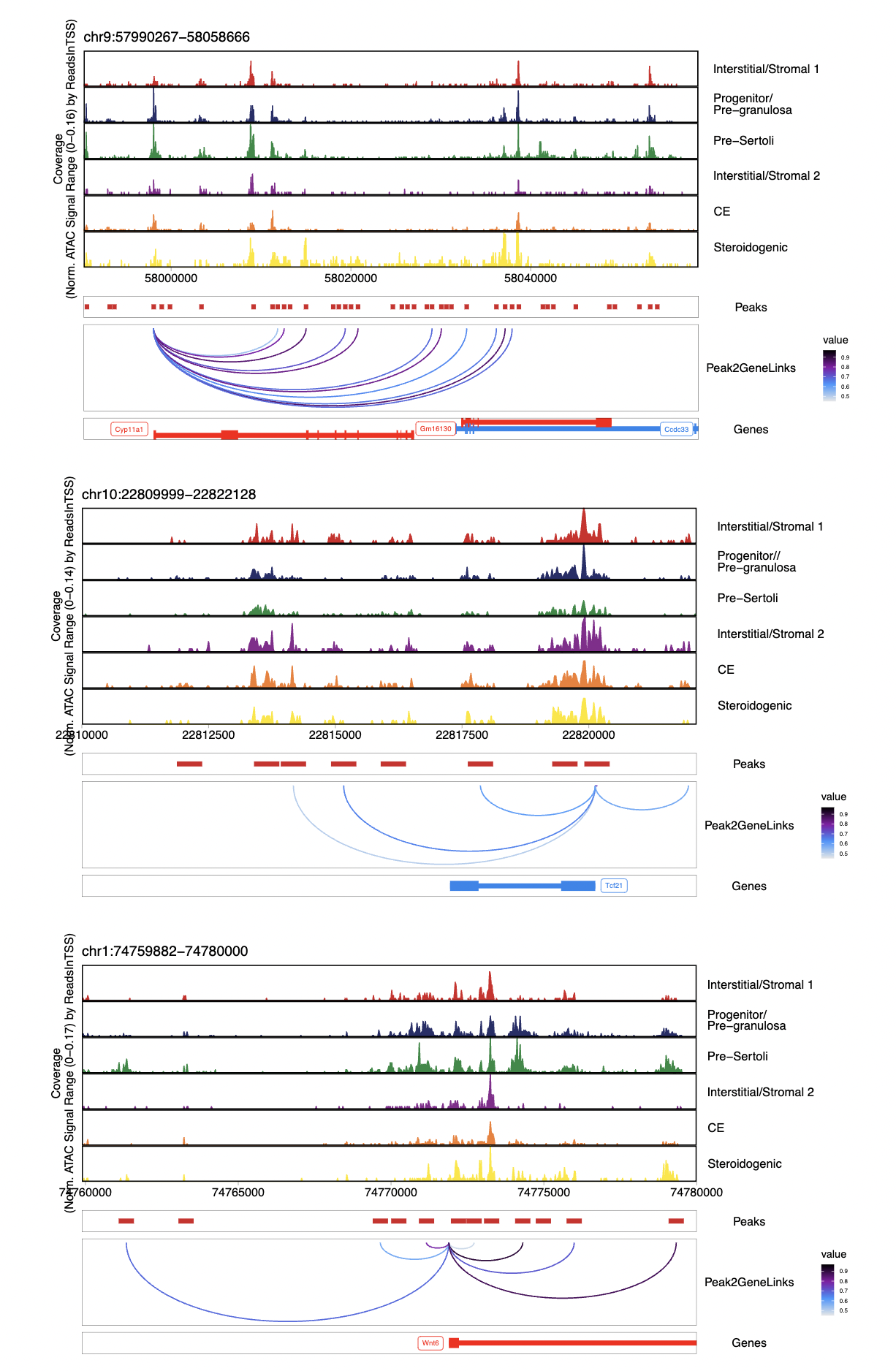
**

**Supplementary Figure S6. Peak-to-gene examples associated with cell type-specific regulators, related to Figure 4.**

Aggregated scATAC-seq profiles showing peak-to-gene links to the *Cyp11a1* (top), *Tcf21* (middle) and *Wnt6* (bottom) loci.

**
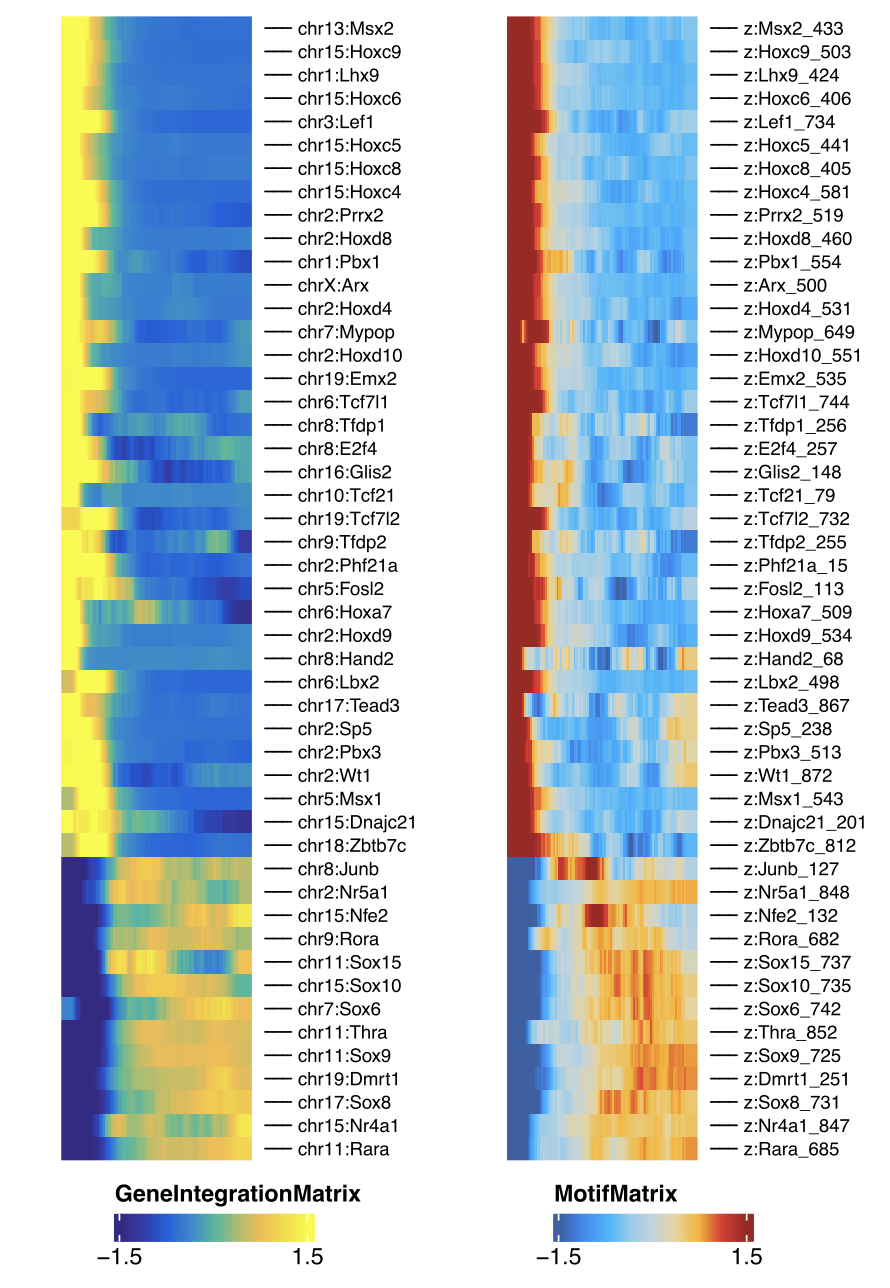
**

**Supplementary Figure S7. Transient patterns of transcription factor expression and regulatory elements during Sertoli cell differentiation, related to Figure 5.**

Heatmap showing dynamic gene expression (left) and transcription factor (TF) motif activity (right) of indicated TFs along pseudotime for gene-motif pairs of the Sertoli cell differentiation trajectory.

**
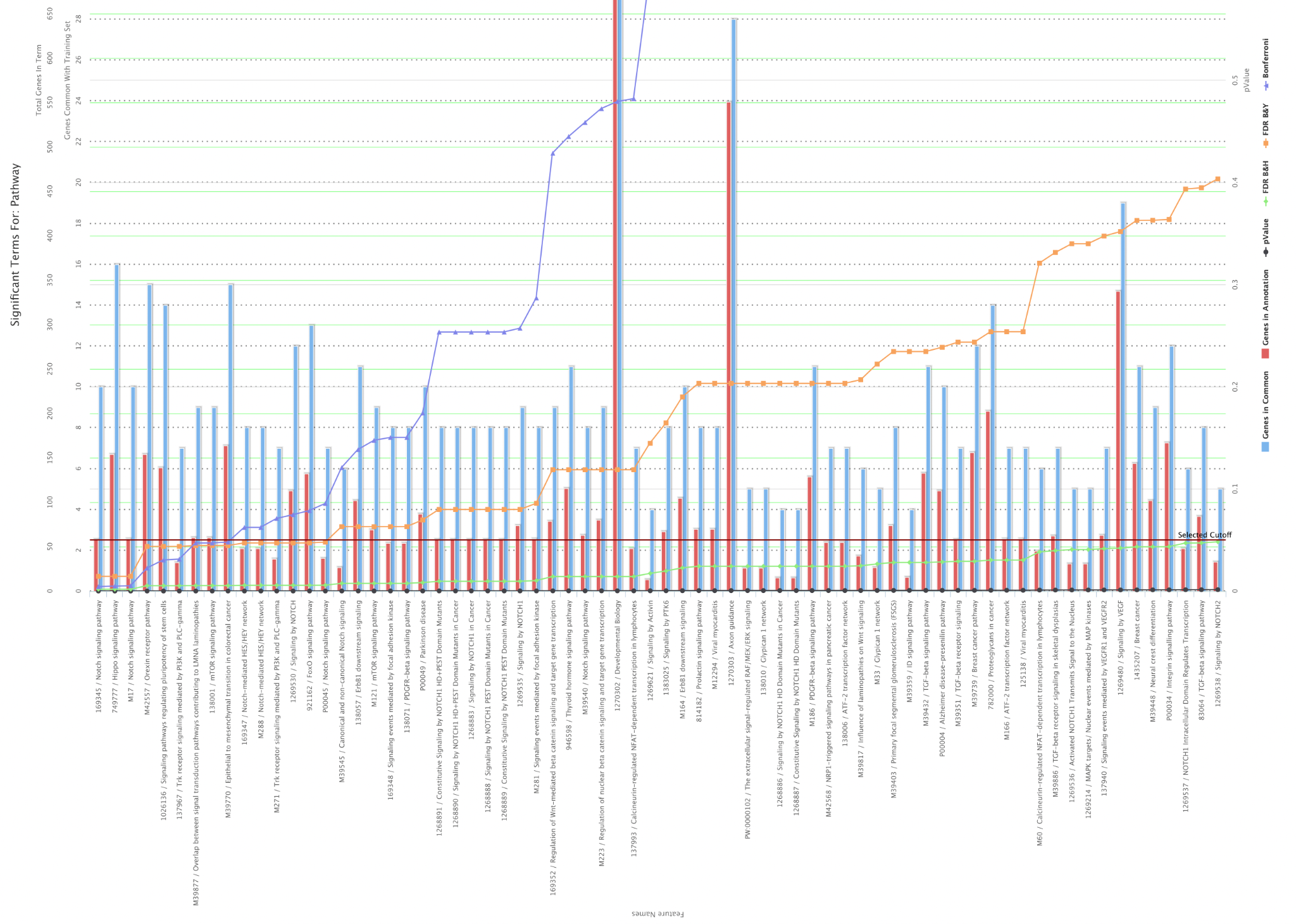
**

**Supplementary Figure S8. Toppgene analysis of peaks along the Sertoli cell differentiation, related to Figure 5.**

Top results from the Toppgene analysis showing the terms associated with the 682 peaks at distal intergenic regions in Supplementary Table S4.

**
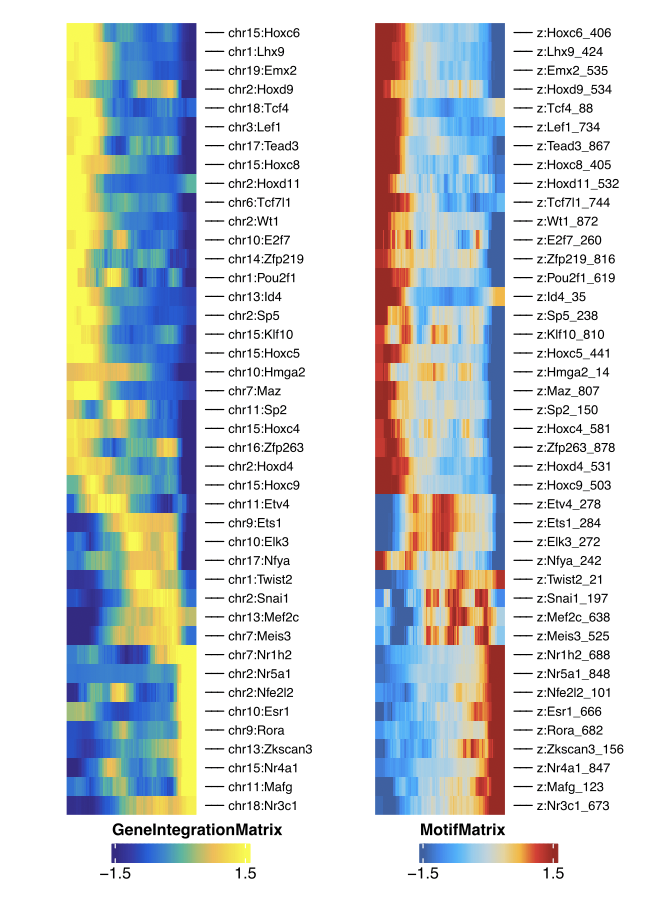
**

**Supplementary Figure S9. Transient patterns of transcription factor expression and regulatory elements during steroidogenic cell differentiation, related to Figure 6.**

Heatmap showing dynamic gene expression (left) and transcription factor (TF) motif activity (right) of indicated TFs along pseudotime for gene-motif pairs of the steroidogenic cell differentiation trajectory.


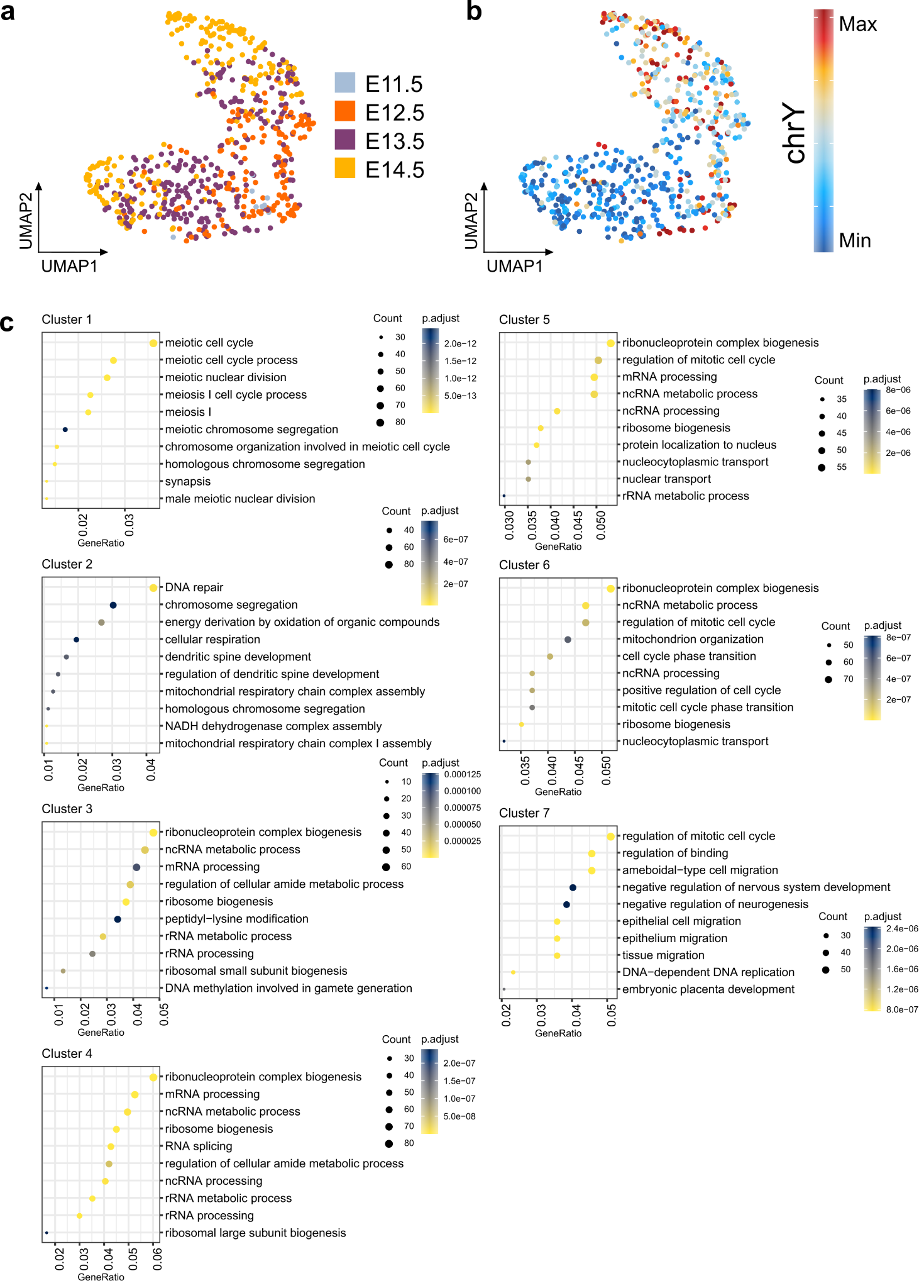


**Supplementary Figure S10. Characterization of peak-to-gene links across germ cell clusters, related to Figure 7.**

**a)** UMAP representation of all germ cells captured from all four time points. Cells are colored by time point and **b)** ChrY score. **c)** Top results from the Gene Ontology (GO) enrichment test showing the terms associated with peak-to-gene links in each cluster identified in Figure 7e.
